# Supplementary material for: A Critical View of the Application of the APEX Software (Aqueous Photochemistry of Environmentally-Occurring Xenobiotics) to Predict Photoreaction Kinetics in Surface Freshwaters
Source: Molecules. 2019 Dec 18;25(1):9. doi: 10.3390/molecules25010009 (PMC7017383; doi:10.3390/molecules25010009)
Supplement: Supplementary file 1 [file molecules-25-00009-s001.zip › 3.2.4_gcc-4.4.0.zip!/3.2.4_gcc-4.4.0/3.2.4_gcc-4.4.0/doc/octave/html/interpreter/Simple-Examples.html]

Simple Examples - Untitled


Next: Conventions,
Previous: Running Octave,
Up: Introduction


---

### 1.2 Simple Examples

The following chapters describe all of Octave's features in detail, but
before doing that, it might be helpful to give a sampling of some of its
capabilities.

If you are new to Octave, I recommend that you try these examples to
begin learning Octave by using it. Lines marked with octave:13>
are lines you type, ending each with a carriage return. Octave will
respond with an answer, or by displaying a graph.

#### 1.2.1 Elementary Calculations

Octave can easily be used for basic numerical calculations. Octave
knows about arithmetic operations (+,-,\*,/), exponentiation (^),
natural logarithms/exponents (log, exp), and the trigonometric
functions (sin, cos, ...). Moreover, Octave calculations work
on real or imaginary numbers (i,j). In addition, some mathematical
constants such as the base of the natural logarithm (e) and the ratio
of a circle's circumference to its diameter (pi) are pre-defined.

For example, to verify Euler's Identity,

```
      i*pi
     e     = -1
```

type the following which will evaluate to `-1` within the
tolerance of the calculation.

```
     octave:1> exp(i*pi)
```

#### 1.2.2 Creating a Matrix

Vectors and matrices are the basic building blocks for numerical analysis.
To create a new matrix and store it in a variable so that you can
refer to it later, type the command

```
     octave:1> A = [ 1, 1, 2; 3, 5, 8; 13, 21, 34 ]
```

Octave will respond by printing the matrix in neatly aligned columns.
Octave uses a comma or space to separate entries in a row, and a
semicolon or carriage return to separate one row from the next.
Ending a command with a semicolon tells Octave not to print the result
of the command. For example,

```
     octave:2> B = rand (3, 2);
```

will create a 3 row, 2 column matrix with each element set to a random
value between zero and one.

To display the value of a variable, simply type the name of the
variable at the prompt. For example, to display the value stored in the
matrix `B`, type the command

```
     octave:3> B
```

#### 1.2.3 Matrix Arithmetic

Octave has a convenient operator notation for performing matrix
arithmetic. For example, to multiply the matrix `A` by a scalar
value, type the command

```
     octave:4> 2 * A
```

To multiply the two matrices `A` and `B`, type the command

```
     octave:5> A * B
```

and to form the matrix product
`transpose (A) * A`,
type the command

```
     octave:6> A' * A
```

#### 1.2.4 Solving Systems of Linear Equations

Systems of linear equations are ubiquitous in numerical analysis.
To solve the set of linear equations `A`x `= b`,
use the left division operator, \:

```
     x = A \ b
```

This is conceptually equivalent to
`inv (a) * b`,
but avoids computing the inverse of a matrix directly.

If the coefficient matrix is singular, Octave will print a warning
message and compute a minimum norm solution.

A simple example comes from chemistry and the need to obtain balanced
chemical equations. Consider the burning of hydrogen and oxygen to
produce water.

```
     H2 + O2 --> H2O
```

The equation above is not accurate. The Law of Conservation of Mass requires
that the number of molecules of each type balance on the left- and right-hand
sides of the equation. Writing the variable overall reaction with
individual equations for hydrogen and oxygen one finds:

```
     x1*H2 + x2*O2 --> H2O
     H: 2*x1 + 0*x2 --> 2
     O: 0*x1 + 2*x2 --> 1
```

The solution in Octave is found in just three steps.

```
     octave:1> A = [ 2, 0; 0, 2 ];
     octave:2> b = [ 2; 1 ];
     octave:3> x = A \ b
```

#### 1.2.5 Integrating Differential Equations

Octave has built-in functions for solving nonlinear differential
equations of the form

```
     dx
     -- = f (x, t)
     dt
```

with the initial condition

```
     x(t = t0) = x0
```

For Octave to integrate equations of this form, you must first provide a
definition of the function
`f(x,t)`.
This is straightforward, and may be accomplished by entering the
function body directly on the command line. For example, the following
commands define the right-hand side function for an interesting pair of
nonlinear differential equations. Note that while you are entering a
function, Octave responds with a different prompt, to indicate that it
is waiting for you to complete your input.

```
     octave:1> function xdot = f (x, t)
     >
     >  r = 0.25;
     >  k = 1.4;
     >  a = 1.5;
     >  b = 0.16;
     >  c = 0.9;
     >  d = 0.8;
     >
     >  xdot(1) = r*x(1)*(1 - x(1)/k) - a*x(1)*x(2)/(1 + b*x(1));
     >  xdot(2) = c*a*x(1)*x(2)/(1 + b*x(1)) - d*x(2);
     >
     > endfunction
```

Given the initial condition

```
     octave:2> x0 = [1; 2];
```

and the set of output times as a column vector (note that the first
output time corresponds to the initial condition given above)

```
     octave:3> t = linspace (0, 50, 200)';
```

it is easy to integrate the set of differential equations:

```
     octave:4> x = lsode ("f", x0, t);
```

The function `lsode` uses the Livermore Solver for Ordinary
Differential Equations, described in A. C. Hindmarsh, ODEPACK, a
Systematized Collection of ODE Solvers, in: Scientific Computing, R. S.
Stepleman et al. (Eds.), North-Holland, Amsterdam, 1983, pages 55–64.

#### 1.2.6 Producing Graphical Output

To display the solution of the previous example graphically, use the
command

```
     octave:1> plot (t, x)
```

If you are using a graphical user interface, Octave will automatically create
a separate window to display the plot.

To save a plot once it has been displayed on the screen, use the print
command. For example,

```
     print -deps foo.eps
```

will create a file called foo.eps that contains a rendering of
the current plot in Encapsulated PostScript format. The command

```
     help print
```

explains more options for the `print` command and provides a list
of additional output file formats.

#### 1.2.7 Editing What You Have Typed

At the Octave prompt, you can recall, edit, and reissue previous
commands using Emacs- or vi-style editing commands. The default
keybindings use Emacs-style commands. For example, to recall the
previous command, press `Control-p` (written `C-p` for
short). Doing this will normally bring back the previous line of input.
`C-n` will bring up the next line of input, `C-b` will move
the cursor backward on the line, `C-f` will move the cursor forward
on the line, etc.

A complete description of the command line editing capability is given
in this manual in Command Line Editing.

#### 1.2.8 Help and Documentation

Octave has an extensive help facility. The same documentation that is
available in printed form is also available from the Octave prompt,
because both forms of the documentation are created from the same input
file.

In order to get good help you first need to know the name of the command
that you want to use. This name of the function may not always be
obvious, but a good place to start is to just type `help`.
This will show you all the operators, reserved words, functions,
built-in variables, and function files. An alternative is to search the
documentation using the `lookfor` function. This function is
described in Getting Help.

Once you know the name of the function you wish to use, you can get more
help on the function by simply including the name as an argument to help.
For example,

```
     help plot
```

will display the help text for the `plot` function.

Octave sends output that is too long to fit on one screen through a
pager like `less` or `more`. Type a <RET> to advance one
line, a <SPC> to advance one page, and <q> to exit the pager.

The part of Octave's help facility that allows you to read the complete
text of the printed manual from within Octave normally uses a separate
program called Info. When you invoke Info you will be put into a menu
driven program that contains the entire Octave manual. Help for using
Info is provided in this manual in Getting Help.
